# How Can Genomic Tools Contribute to the Conservation of Endangered Organisms

**DOI:** 10.1155/2016/4712487

**Published:** 2016-11-15

**Authors:** Cino Pertoldi, Ettore Randi, Aritz Ruiz-González, Philippine Vergeer, Joop Ouborg

**Affiliations:** ^1^Department of Chemistry and Bioscience, Aalborg University, Aalborg Zoo, Mølleparkvej 63, 9000 Aalborg, Denmark; ^2^Istituto Superiore per la Protezione e la Ricerca Ambientale ISPRA, Roma, Italy; ^3^Department of Zoology and Animal Cell Biology, University of the Basque Country, UPV/EHU, Vitoria-Gasteiz, Spain; ^4^Nature Conservation and Plant Ecology Group, Wageningen University, Wageningen, Netherlands; ^5^Experimentele Planten Ecologie, IWWR, Radboud Universiteit, Nijmegen, Netherlands

Conservation biologists have realized the urgent need for genomic tools and interdisciplinary approaches to help understand the loss of biodiversity. Conservation genetics has been successful in highlighting the roles of evolutionary and population genetics for biodiversity conservation, yet several critical issues remain. Rapid development of new sequencing techniques means conservation genomics can now help answer some of the crucial issues that conservation genetics was able to highlight but not to resolve. These include a better understanding of outbreeding and inbreeding depression and the extent to which estimates of heterozygosity accurately reflect quantitative trait variation and fitness. Conservation genomics can help resolve taxonomic uncertainties, contribute to designation of evolutionary significant units and ecotypes, and identify contemporary versus historic patterns of hybridization. The definition of a species is nevertheless a normative concept and cannot be resolved by genomics alone. All living organisms are faced with a multitude of challenges in their natural environment, such as climate change, diseases, predation, competition, and habitat disturbance. In the short term, animals and plants can acclimatize to shifting environmental conditions by developing and expressing particular traits in response to local environmental conditions (phenotypic plasticity). Organisms can also react to the shifting environment by dispersal; however this option is not always available when, for example, the landscape is too fragmented. The last type of response is evolution via genetic selection leading to adaptation. The persistence of species and populations depends however on the initial response to the shifts in the environment.

The genome of a species contains signatures of these responses that may be studied with genetic markers. We are interested in filling gaps in the knowledge about past demographic history of organisms and the factors that ultimately shape genomic variation in populations. For this we use the very latest, innovative techniques in next generation sequencing. The incorporation of technological developments in molecular biology and the ongoing development of genomic tools, like SNPs and next generation sequencing, and genomic-based approaches, like full genome scans and gene-expression pattern analysis, make it possible to address questions that until now were hard to tackle. There is an urgent need for empirical studies on nonmodel organisms which can contribute to the emerging disciplines of population genomics and landscape genomics. Such studies are necessary if we want to show how these advances in molecular techniques and approaches allow conservation genetics to make a big leap forward. Incorporating genetics and genomics into nature conservation will highlight the following:

- Genomic consequences of inbreeding
- Inbreeding by environment interaction
- Genomic and epigenomic consequences of outbreeding
- Genomic and epigenomic mechanisms of phenotypic plasticity
- Transcriptome, metabolomics, and proteomic techniques applied to conservation biology
- The emerging discipline of landscape genomics; detection of signature of selection using genomic techniques

All the results from the accepted papers have greatly contributed to the special issue. To optimize the use of limited resources in conservation biology and conservation genomics and minimize the need to sample and disturb wild species (often those most in need of investigation are the most sensitive to disturbance), we recommend increased public sharing of resources including genetic markers, data, and analytical tools and improved professional recognition for the publication of genomic resources.

